# Preservice Preschool Teachers' Responses to Bullying Scenarios: The Roles of Years of Study and Empathy

**DOI:** 10.3389/fpsyg.2018.00175

**Published:** 2018-02-20

**Authors:** Heqing Huang, Yanchun Liu, Yulu Chen

**Affiliations:** ^1^Laboratory of Infant and Child Learning and Development, College of Preschool Education, Capital Normal University, Beijing, China; ^2^Department of Psychology, College of Educational Science, Hubei Normal University, Huangshi, China; ^3^Department of Psychology, Teachers' College, Beijing Union University, Chaoyang, China

**Keywords:** preservice teachers, bullying, empathic concern, personal distress, preschool teachers, empathy

## Abstract

The present study is aim to examine whether preservice preschool teachers' respond differently to physical, verbal and relational bullying, and how their years of study and trait empathy related their responses. There were 242 preservice teachers in the present study. Empathy was measured with the self-report Interpersonal Reactive Index; the Bullying Attitude Questionnaire was used to assess their perceptions of incident seriousness, their sympathy toward the victim of the bullying, and their possibility to intervene in the situation. The results revealed that the participants responded most to physical bullying and least to relational bullying. Interestingly, responses to relational bullying tended to decrease during the university period. The empathy dimensions played different roles in the participants' responses to bullying, while cognitive empathy have little relationship with the participants' responses to bullying, emotional empathy played a more complex role in the participants' responses to bullying: empathic concerns moderated the relationship between years of study and responses to bullying, and personal distress negatively predicted the participants' responses to all types of bullying. The implications for bullying intervention and the suggestion for teacher education are discussed.

## Introduction

### Teachers' responses to bullying

Bullying is the aggression which aims to harm, humiliate, intimidate, or isolate a weaker person (Smith et al., 2002; Hymel et al., 2015). Most research concerning bullying has focus their emphasis on school children, while only limited empirical research pay attention to the emergence and development of bullying in early childhood years (Vlachou et al., 2011; Chan and Wong, 2015). Results from a limited number of studies suggest that aggression and bullying are relatively frequent in many preschool classrooms (Murray-Close and Crick, 2006; Camodeca et al., 2015; see Vlachou et al., 2011).

Bullying in preschool may take different forms: overt bullying, for example the physical or verbal bullying (Olweus, 1993), and indirect bullying, typically the relational bullying (Crick and Grotpeter, 1995). While physical and verbal bullying dominate preschoolers' bullying behavior, relational bullying increases gradually with social cognition development (Humphrey, 2013). During school period, relational bullying has been found to increase while physical and verbal bullying decreases (Scheithauer et al., 2010). In older children, relational bullying behaviors are more subtle and ambiguous and are consequently more difficult to detect (Bauman and Del Rio, 2006; Splett et al., 2015; Brion-Meisels and Garnett, 2016). The manifestation of relational aggression and bullying in the preschool years is similar to that of school-age children in many ways. However, the bullying in early childhood includes some unique features. When engage in relationally bullying, the preschoolers usually tend to do so in more direct and simple ways which involve a current situation, for example, declaring that unless a peer gives her/him a toy, she/he will not be the peer's friend.

For both the bullies and the victims, the experience of bullying has been linked to a range of concurrent and long-term negative outcomes including academic, social, emotional, and behavioral problems (Golmaryami et al., 2016). Intervening in bullying as early as possible is critical (Saracho, 2017). Preschools play important roles in children's social development. As the first and the closest niche beyond their home environment, children's difficulties in social interactions with peers are primarily detected by their teachers. Timely identification and elimination of these problems at this young age can help avoiding their escalation in later years and minimize their negative affect on children's social and emotional development while promoting their successful adaptation to school (Vlachou et al., 2011).

Teachers play important roles in intervening in early bullying. As teachers are the formal authority figures in the classroom, they often assume a central role in managing bullying (Wang et al., 2015; Campaert et al., 2017). When bullying happens in schools, teachers can carried out immediate interventions to help stop bullying (Veenstra et al., 2014; Camodeca et al., 2015). Teachers' responses to bullying incidents will influence the likelihood of children' future bullying behaviors (Hektner and Swenson, 2012). Moreover, the interventional strategies teachers employ can affect students' bullying behavior (e.g., Byers et al., 2011). However, most research indicates that teachers do not respond timely and effectively to children's bullying incidents, for example, Oldenburg et al. pilot study (2016) reported that teachers in Dutch primary school were unprepared to tackle bullying events; they gave incomplete knowledge of bullying, did not recognize the victims in their classrooms, and had limited strategies to find out about bullying. Bauman and Del Rio (2006) indicated that preservice teachers tended to respond more to direct forms of bullying, for example physical and verbal bullying, than to indirect forms like relational bullying. However, it remains unknown whether and how preschool teachers respond to bullying behaviors in preschool settings. No research have indicated the relationship between the teachers' responses to bullying and the time they spend in education. However, as previous studies have indicated that expert teachers are more experienced in class management and problem-resolving (Livingston and Borko, 1989), the preschool teachers are expected to respond more positively to the children's bullying with the increase of their time and experience in preschool education. In the present study, we focused on the preservice stage of teacher development when they form their professional attitudes and skills toward young children and their teaching career (Clark et al., 2015). Moreover, it is important to point out, although the time spending in education may have high correlation with the participants' age, they are two distinct concepts. Age is an index of maturity, while the years of study is the index of the preservice teachers' experience of preschool education. In the present study we use the years of study as the index of the preservice teachers' experience in preschool education.

### The role of empathy in preschool teachers' reaction to bullying behaviors

Empathy is the capacity to feel and understand what others feel, is an important and complex interpersonal function (Huang and Su, 2014; Main et al., 2017). It is widely accepted that empathy is a multifaceted construct which involves at least an emotional component and a cognitive component (Preston and de Waal, 2002; Decety, 2011). The cognitive component of empathy refers to the ability to infer and understand others' emotion relies on attributing emotional states onto others (Decety, 2011), and some researcher believe cognitive empathy contains the ability to walk in shoes of others, who may be real persons or fictional characters (Davis, 1983; Huang and Su, 2014). The emotional component of empathy refers to experiencing the feelings and emotional states of others without confusion between oneself and others (Chiu and Yeh, 2017). Some researchers underscore the emotional nature of empathy and furthermore differentiated between two empathy-related reactions: empathic concern and personal distress (Eisenberg et al., 1989; Neumann et al., 2016; Buck et al., 2017). Empathic concern, which is based on the understanding of another person's emotional or living situation, is essentially an other-oriented emotional reaction; consequently, empathic concern motivates individuals to take prosocial actions. Whereas, personal distress, which is defined as aversive emotional over-arousal and self-oriented. If an individual feels personal distress, he or she is more likely to escape a situation, physically or psychologically, than to help the individual in the situation (Eisenberg et al., 1989; Organizer and Goode, 2008).

Empathy has various relationships to teachers' work, for example, empathy affect the teachers' communication skills and the students' development outcomes (Cooper et al., 2000; Ahmetoglu and Acar, 2016). However previous studies did not distinguish the effects of teachers' cognitive and emotional empathy in educational context. Research from related areas suggested that cognitive and emotional empathy may play different roles in teachers' responses to children's bullying. On the one hand, as a large number of research have supported a positive role of cognitive empathy in a wide range of human social function (see Huang and Su, 2014), especially the altruistic and helping behavior (Underwood and Moore, 1982), cognitive empathy may related with the teachers' positive responses to bullying. It is reasonable that the more a teacher walks in the shoes of the child involving in bullying behavior, the less likely that he or she will neglect or negatively respond to the bullying incident. On the other hand, emotional empathy plays an important and complex role in teachers' responses to children's bullying, and empathic concern and personal distress may have different relationship with their responses. When exposed to bullying incidents, teachers may feel various emotions (e.g., sad, upset, perturbed) that lead them to response to the bullying, and the teachers have different tendencies of empathy are assumed to have different responding pattern to bullying. For example the teacher with personal distress may scold the crying child, or escape the situation. Alternatively, teachers with empathetic concern may also regulate their feelings and orient to the child in the bullying situation. Teachers may feel compassion and take steps to minimize the likelihood of bullying. Moreover, researcher also pointed out that some potential variables, for example emotional regulation, may mediate the relation between empathy and the helping behavior (Lockwood et al., 2014). However, few studies have investigated the role of empathy in the responses to bullying behavior of teachers and those who are plan to become teachers, even though empathy has been documented as a necessary disposition for educators to facilitate positive interactions among students (Boyer, 2010). According to the previous studies, the other-oriented and the self-oriented components may play different roles in the preschool teachers' responses to bullying behavior. Therefore in the present study, we hypothesis that empathic concern, the other-oriented dimension of empathy, related to the preschool teachers' positive responses to bullying; while personal distress, which is the self-oriented dimension of empathy, tend to be related with preschool teachers' negative responses to bullying.

Moreover, the relationship between empathy and the preschool teachers' responses to bullying may change with the time which they spend in preschool education. As Rushton et al. (2007) proposed, personality can be shaped by one's career. As a personality trait, empathy and its components are dynamic and can be affected by the environment. Recent research indicated that, in certain professions, empathy and its components showed a decreasing trend over time. Neumann et al. (2011) examined the empathy of students from various majors and found that medical students' cognitive empathy significantly decreased in their third year. In the preschool education profession, which is also a helping profession, it is unclear whether empathy will show a similar decline with career experience.

Examining preschool teachers' responses to bullying behavior and the related factors underlying their responses is of great importance. Our questions is how preschool teachers with different studying years respond to different types of bullying behaviors, and what roles the empathy dimensions play in these responses. Based on previous research, we hypothesized that preservice preschool teachers respond differently to physical, verbal, and relational bullying, with the least response to the last form. We also proposed that preschool teachers' responses to bullying may positively correlated with time which they studying preschool education. Moreover, we proposed that the different dimensions of empathy play different roles in shaping preservice preschool teachers' responses to the three types of bullying: both the two components of cognitive empathy positively related with teachers' responses to bullying, and the two components of emotional empathy, empathic concern and personal distress, may play different roles in teachers' response to bullying. The two main aims of this study were to: (a) describe the characteristics of the preservice preschool teachers' responses to children's bullying behaviors; and (b) examine the roles of different components of empathy in responses to bullying behaviors.

## Method

### Participants

Undergraduate students attending a preschool college at a typical Chinese university comprised the sample. In China, undergraduate students majoring in preschool education spend four years at university. The last school term is spent in practice in preschools and completing a thesis, while earlier regular school terms consist of theory courses and 1 week per school term on practicing in the preschools. According to previous data of this university, about 90% of graduating students take preschool teacher positions.

In the present study, all 242 participants were female with ages ranging from 19 to 21 years, with a mean age of 20.25 (*SD* = 1.31). Among them, 29.34% (*n* = 71, *M*_age_ = 18.80, *SD*_age_ = 0.44) were freshmen; 28.51% (*n* = 69, *M*_age_ = 19.92, *SD*_age_ = 0.49) were sophomores; 22.31% (*n* = 54, *M*_age_ = 20.90, *SD*_age_ = 0.52) were juniors; and 19.83% (*n* = 48, *M*_age_ = 22.05, *SD*_age_ = 0.55) were seniors. The participants were all females, because males only account for 3-5% of the populations of both preschool and in-service teachers (Sak et al., 2012).

Most participants came from families of middle socioeconomic status (*M* = 5.43, *SD* = 1.46; 5 = ¥100,000–200,000, 6 = ¥200,000–300,000) (Zhang and Shin, 2015). Specifically, 64.26% of the participants came from families whose income ranged from ¥100,000 to ¥200,000, 26.05% were from families with income between ¥200,000 and ¥300,000, 3.46% came from families with income higher than ¥300,000, and 6.23% came from families with incomes less than ¥100,000.

### Procedure

On data collection days, participants were instructed to complete a serial of anonymous questionnaires in a classroom setting. The researchers stayed in the classroom and were available to answer the possible questions. The students were rewarded with appropriate credit for their attending the research. Participation rates within each classroom ranged from 95.54 to 100% (*M*_*age*_ = 98.13%).

### Measures

#### The Chinese variant of the interpersonal reactivity index (IRI-C)

The Chinese variant of the Interpersonal Reactivity Index (IRI; Davis, 1983) developed by Huang and Su (2014) was used to assess the empathy of the participants. The IRI-C is a 28-item self-report questionnaire which measures different dimensions of empathy; it comprises four 7-item subscales (empathic concern, personal distress, fantasy, and perspective taking). The empathic concern subscale was designed to examine one's capacity to experience feelings of warmth, compassion, and concern to another person in need (e.g., “I often have tender, concerned feelings for people less fortunate than me”). The personal distress subscale was designed to examine an individual's own negative emotions as they respond to stressful interpersonal situations (e.g., a reversed item “When I see someone get hurt, I tend to remain calm”). The perspective taking subscale assesses unplanned attempts to adopt others' points of view (e.g., “I really get involved with the feelings of the characters in a novel.”). And the fantasy subscale was designed to examine the likelihood that an individual identifies with a fictional character (e.g., “I really get involved with the feelings of the characters in a novel.”). The participants were asked to report on a 5-point Likert scale ranging from 1 (does not agree with me) to 5 (agrees with me very well). All of the subscales demonstrated adequate internal reliability with alpha values of 0.77, 0.82, 0.79, and 0.81 respectively. According to Davis (1983), the subscales of empathic concern and personal distress involve the emotional aspect of empathy, the subscales of perspective taking and fantasy reflect the cognitive aspect of empathy.

#### Preservice teachers' reactions to preschoolers' bullying behaviors

We adapted a questionnaire used in Bauman and Del Rio's (2006) study and this questionnaire consisted of six vignettes, in which the readers will witness an incident taking place in an ambiguous situation. Among the vignettes, two describe incidents of physical bullying, two portray a verbal bullying scenario, and two present relational bullying situations. Each vignette shows two children of about 3–5 years old, with one acting as the bully and the other acting as the victim. The ambiguous environment described in the vignette can be the preschool or the community. In the vignettes, the children's gender and ethnicity are neutral and ambiguous. After each vignette, the participants were required to answer three questions on a 5-point Likert scale, which were: to what extent they evaluate the incident is serious, to what extent they feel sympathy for the bullying victim, and their likelihood to intervene the bullying incident.

The method of vignettes are widely used in awareness and attitudinal research, and have advantages in eliminating social desirability effects and potential observer effects, some researchers believed the vignettes are more closely approximate the decision-making situation in real life than would the self-report methods such as interviews or questionnaires, and consequently advanced in the external validity (Barter and Renold, 2000).

Inter-rater reliability of the vignettes questionnaire was established using Cohen's kappa. All of the subjects' responses to the six bullying vignettes were coded by the first author. A second coder, who was blind to the purpose of the present study, was trained and coded data from 50 participants (~21%), and the Cohen's kappa was 0.87.

## Results

The descriptive analyses of the participants' dimensions of empathy and their three reactions to different types of bullying are shown in Table 1. In addition to the descriptive statistics, three sets of analyses were conducted. First, we used repeated measures multivariate analyses of variance (MANOVA) to compare the degrees of reactions to different bullying behaviors among preservice teachers with 1–4 years of study. Secondly, one-way analyses of variance (ANOVAs) were conducted to determine whether preservice teachers' dimensions of trait empathy differed across their years of study. Lastly, we carried out correlation and regression analyses to investigate the roles of studying years and empathy in preschool teachers' reactions to different forms of bullying.

**Table 1:** Descriptive analyses of the preschool teachers' dimensions of empathy, and three reactions to the bullying.

		**The 1st year of study *n* = 71**	**The 2nd year of study *n* = 69**	**The 3rd year of study *n* = 54**	**The 4th year of study *n* = 48**	**Total *N* = 242**	**ANOVAs**
Teachers' reaction to physic bullying	Seriousness perception	9.01 (1.26)	8.81 (1.50)	8.59 (1.46)	8.74 (1.54)	8.81 (1.43)	*F*_(3, 236)_ = 1.59, *p* = 0.19
	Empathy for the victim	8.25 (1.71)	7.58 (1.84)	7.57 (2.01)	7.63 (2.35)	7.28 (1.75)	*F*_(3, 236)_ = 2.42, *p* = 0.07
	Likelihood of intervention	8.79 (1.53)	8.61 (1.50)	8.85 (1.17)	8.84 (1.49)	7.55 (1.68)	*F*_(3, 234)_ = 0.47, *p* = 0.70
Reaction to verbal bullying	Seriousness perception	7.38 (1.74)	7.12 (1.83)	6.65 (1.85)	7.00 (2.18)	7.07 (1.88)	*F*_(3, 236)_ = 0.93, *p* = 0.43
	Empathy for the victim	7.72 (1.45)	7.09 (1.70)	6.96 (1.78)	7.29 (2.11)	7.28 (1.75)	*F*_(3, 236)_ = 1.92, *p* = 0.13
	Likelihood of intervention	7.65 (1.73)	7.72 (1.79)	7.35 (1.52)	7.71 (1.66)	7.55 (1.68)	*F*_(3, 236)_ = 0.59, *p* = 0.90
Reaction to relationship bullying	Seriousness perception	8.09 (1.60)	7.35 (1.86)	7.05 (1.83)	6.96 (2.02)	7.43 (1.85)	*F*_(3, 233)_ = 4.83, *p* = 0.005
	Empathy for the victim	8.03 (1.63)	7.07 (1.71)	6.75 (1.96)	6.85 (2.10)	7.24 (1.89)	*F*_(3, 233)_ = 6.47, *p* = 0.001
	Likelihood of intervention	8.10 (1.91)	7.62 (1.89)	7.49 (1.44)	7.51 (1.66)	7.71 (1.77)	*F*_(3, 233)_ = 1.65, *p* = 0.18
Dimensions of empathy	Empathic concern	3.66 (0.46)	3.67 (0.47)	3.45 (0.58)	3.67 (0.47)	3.64 (0.50)	*F*_(3, 233)_ = 4.21, *p* = 0.006
	Personal distress	2.88 (0.49)	2.98 (0.43)	3.10 (0.62)	3.00 (0.52)	2.96 (0.52)	*F*_(3, 233)_ = 2.42, *p* = 0.067
	Perspective taking	1.90 (0.52)	1.86 (0.36)	1.68 (0.54)	2.04 (0.49)	1.87 (0.43)	*F*_(3, 232)_ = 4.46, *p* = 0.005
	Fantasy	1.45 (0.45)	1.54 (0.57)	1.62 (0.51)	2.52 (0.63)	1.53 (0.54)	*F*_(3, 231)_ = 0.98, *p* = 0.404

### Preservice teachers' responses to different bullying behaviors

**Figure 1:**
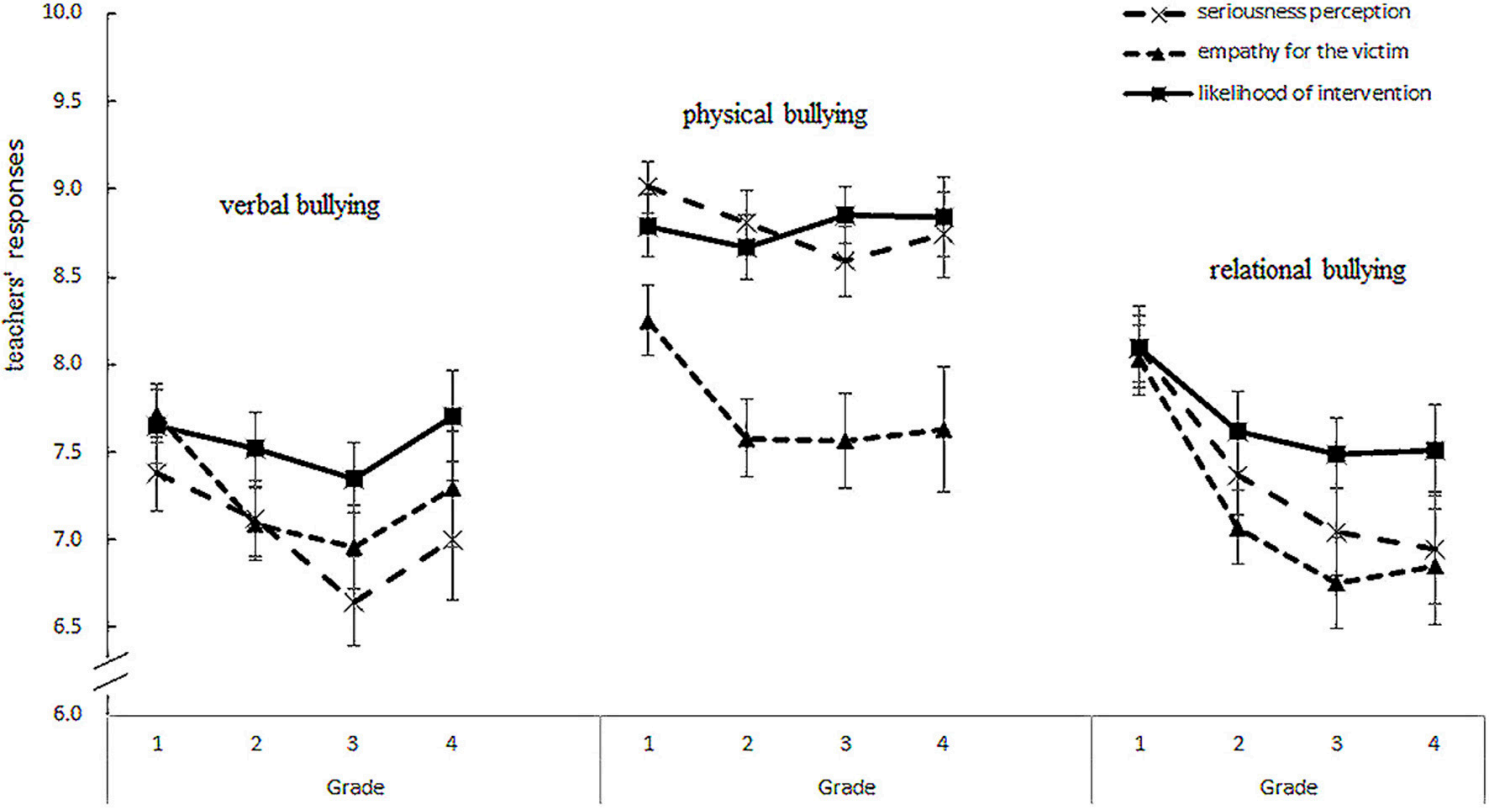
Preservice preschool teachers' responses to three different bullying behaviors.

A 3 (types of bullying behaviors) × 3 (indexes of the participants' responses) × 4 (studying years of participants) repeated measures MANOVA was performed to profile the teachers' responses to bullying. Among the variables, the categories of bullying behavior and the indexes of the participant responses are within-subject variables, and the participants' years of study is the between-subject variable. The results are shown in Figure 1.

We observed a significant main effect of teachers' years of study: *F*_(3, 224)_ = 3.30, *p* = 0.02, $\eta_{p}^{2}$ = 0.04. The Bonferroni *post hoc* test revealed that for all three indexes to all three types of bullying, the preservice teachers in their first studying year responded significantly more than those in their third studying year (*p* = 0.03).

Second, the results revealed a significant interactive effect between bullying type and the preservice teachers' responses indexes. The sphericity assumption for repeated measures MANOVA was violated: Mauchly's *W*_(2)_ = 0.72, $\chi_{\left(2\right)}^{2}$ = 72.35, *p* < 0.001. Because violating this assumption inflates the Type I error rate, we used the Huynh-Feldt epsilon to adjust the degrees of freedom and provide a more accurate Type I error rate (Liu, 2002; Stevens, 2002). The interactive effect between the bullying types and responding indexes was significant, *F*_(4, 221)_ = 35.91, *p* < 0.001, $\eta_{p}^{2}$ = 0.14. Since it can be analyzed from two perspectives, we conducted two sets of simple-effect analyses (see Figure 1).

In the first set, three repeated ANOVAs were conducted, which made the three bullying types within-subject independent variables and the participants' three responding indexes as dependent variables. For all three ANOVAs, the sphericity assumptions for repeated measures MANOVA was accepted: Mauchly's *W*_(2)_ = 0.99, 0.99, 1.00, $\chi_{\left(2\right)}^{2}$ = 1.37, 2.99, 0.64 for the seriousness evaluation, sympathy for the victim, and likelihood of intervening, respectively (all *p*s > 0.05). As a result, we did not need to adjust the degrees of freedom. The result indicated that for each of the three response indexes, there were significant differences between the three bullying types: for seriousness evaluation, *F*_(2, 456)_ = 156.41, *p* < 0.001, $\eta_{p}^{2}$ = 0.41; for sympathy to the victims, *F*_(2, 458)_ = 21.34, *p* < 0.001, $\eta_{p}^{2}$ = 0.09; and for the likelihood of intervening, *F*_(2, 458)_ = 106.07, *p* < 0.001, $\eta_{p}^{2}$ = 0.32.

The *post hoc* tests revealed that for the seriousness evaluation, preservice teachers evaluated physical bullying as significantly more serious than verbal bullying (*p* < 0.001) and relational bullying (*p* < 0.001). Moreover, they also considered verbal bullying more serious than rational bullying (*p* = 0.002); for their sympathy to the victims, preservice teachers showed significantly more sympathy for victims of physical bullying than verbal and relational bullying (*p*s < 0.001). For the likelihood of intervening, preservice teachers were more likely to intervene in physical bullying than verbal or relational bullying (*p* < 0.001). The set of ANOVAs revealed a similar pattern in that preschool teachers responded most strongly to physical bullying and less to relational bullying.

In the second set of simple-effect analyses, three repeated ANOVAs were conducted, with the preservice teachers' three responding indexes as independent variables and the three bullying types as dependent variables. For each ANOVA, the sphericity assumptions for repeated measures ANOVA was violated, Mauchly's *W*_(2)_ = 0.97, 0.90, 0.90, $\chi_{\left(2\right)}^{2}$ = 6.17, 25.86, 25.86 for physical, verbal, and relational bullying, respectively (all *p*s > 0.05), so the degrees of freedom were adjusted with the Huynh-Feldt epsilon. For physical bullying, the degrees of the three responding indexes differed significantly: *F*_(4, 225)_ = 60.74, *p* < 0.001, $\eta_{p}^{2}$ = 0.04. Subsequent pairwise comparisons indicated that the preservice teachers' seriousness evaluation and likelihood of intervening were both significantly higher than their sympathy for the victims. This result implies that preservice preschool teachers always evaluate a physical bullying incident very seriously and take steps to intervene. However, they tend to have less sympathy for the victims. The degrees of the three responding indexes also differed significantly for verbal bullying: *F*_(4, 224)_ = 8.98, *p* < 0.001, $\eta_{p}^{2}$ = 0.04. Pairwise comparisons indicated that the preservice teachers' seriousness evaluation was significantly lower than their sympathy (*p* < 0.05) and likelihood of intervening (*p* < 0.001), and their sympathy was significantly lower than their likelihood of intervening (*p* < 0.05). This suggests that preservice preschool teachers evaluate verbal bullying as less serious, although they tended to sympathize with the victim and were more likely to intervene. Last, for the relational bullying, significant differences were found between the degrees of the three responding indexes: *F*_(4, 225)_ = 60.74, *p* < 0.001, $\eta_{p}^{2}$ = 0.04. Pairwise comparisons revealed that the preservice teachers' likelihood of intervening was significantly higher than the other two indexes (*p*s < 0.001), and their sympathy was significantly lower than the other two indexes (*p*s < 0.001). This suggests that when facing a relational bullying incident, preservice preschool teachers had the highest likelihood of intervening and had the least sympathy for the victim. Overall, the participants had different response patterns to each bullying type.

### Preservice teachers' empathy

To determine the developmental trends of the four components of empathy in preservice preschool teachers, we conducted ANOVAs with the four IRI dimensions as dependent variables and the studying years of the preservice teachers as independent variables. As Table 1 shows, except the dimension of fantasy, preservice teachers with 1–4 years of study differed significantly on all the other three empathy dimensions, they are empathic concern, personal distress, and perspective taking. For empathic concern, *F*_(3, 227)_ = 4.21, *p* < 0.006, $\eta_{p}^{2}$ = 0.05; and Bonferroni *post hoc* testing revealed that the empathic concern of the participants who were in their third year of study was significantly lower than those in other year of study. For perspective taking, *F*_(3, 231)_ = 4.46, *p* = 0.005, $\eta_{p}^{2}$ = 0.05; and Bonferroni *post hoc* testing revealed that there is significant difference between the perspective taking of the participants who were in their third year and those who were in the fourth year of study. There was also a marginal difference of the studying years in the personal distress dimension, *F*_(3, 230)_ = 2.49, *p* = 0.05, $\eta_{p}^{2}$ = 0.03. Bonferroni *post hoc* testing showed that the personal distress of participants in the third studying year was significantly higher than that of the preservice teachers in the first year of study. These results imply that the third year of study is the developmental turning point for both the empathic concern and personal distress in teacher candidates.

### The relationship between teacher empathy and responding to bullying

Pearson correlation analysis suggested various significant correlations between preservice teachers' dimensions of empathy and responding to bullying, which met the preliminary conditions of regression analyses (Table 2). To further examine the roles of studying years and the dimensions of empathy in the participants' responses to bullying behaviors, we conducted nine hierarchy regression analyses using the teachers' responses (the respondent's seriousness evaluation, sympathy for the victim, and the possiblity to intervene) to the physical, verbal, and relational bullying behaviors as the dependent variable in each. We did not include age in the regression equation because the sample consisted solely of undergraduate students, whose years spent on preschool study were in high correlation with their age, and the Pearson correlation analysis suggested that this correlation was as high as 0.925. Consequently, if we controlled participants' age as a covariate, we could not fully assess the effect of preschool education study on the relationship between empathy and bullying response. As Table 3 shows, the first model included the preservice teachers' studying years, the second model included empathic concern and personal distress, and the third model was the product of the *Z* scores of grade and the two empathy dimensions.

**Table 2:** The relationship between the teachers' years of study, dimensions of empathy, and responses to bullying.

	**2**	**3**	**4**	**5**	**6**	**7**	**8**	**9**	**10**	**11**	**12**	**13**	**14**
1 Years of study	0.01	0.14^*^	0.03	0.07	−0.11	−0.11	−0.01	−0.09	−0.12	0.02	−0.23^**^	−0.24^**^	−0.13
2 Empathic concern		0.26^**^	0.58^**^	0.41^**^	0.20^**^	0.34^**^	0.14^*^	0.19^**^	0.29^**^	0.12	0.09	0.19^**^	0.08
3 Personal distress			−0.02	0.33^**^	−0.01	0.14^*^	−0.11	0.04	0.09	−0.09	−0.03	0.06	−0.08
4 Perspective taking				0.17^**^	0.12	0.20^**^	0.09	0.13	0.16^*^	0.13	−0.01	0.07	0.04
5 Fantasy					0.12	0.22^**^	0.11	0.21^**^	0.26^**^	0.10	0.09	0.14^*^	−0.02
6 Verbal_ Seriousness						0.60^**^	0.45^**^	0.58^**^	0.40^**^	0.21^**^	0.61^**^	0.45^**^	0.36^**^
7 Verbal_ Sympathy							0.42^**^	0.44^**^	0.60^**^	0.24^**^	0.49^**^	0.67^**^	0.27^**^
8 Verbal_ Intervention								0.38^**^	0.35^**^	0.60^**^	0.43^**^	0.41^**^	0.68^**^
9 Physical_ Seriousness									0.59^**^	0.54^**^	0.57^**^	0.45^**^	0.35^**^
10 Physical_ Sympathy										0.45^**^	0.42^**^	0.68^**^	0.37^**^
11 Physical_ Intervention											0.33^**^	0.29^**^	0.60^**^
12 Relational_ Seriousness												0.75^**^	0.66^**^
13 Relational_ Sympathy													0.57^**^
14 Relational_ Intervention													

* *p < 0.05*.

** *p < 0.01*.

**Table 3:** Hierarchy regressions predicting the preservice teachers' responses to three different bullying.

		**Model 1**	**Model 2**					**Model 3**								
	**Yos**		**Yos**	**EC**	**PD**	**PT**	**FS**	**Yos**	**EC**	**PD**	**PT**	**FS**	**Yos × EC**	**Yos × PD**	**Yos × PT**	**Yos × FS**
Seriousness Physic		*R*^2^ = 0.01, *F*_(1, 219)_ = 2.09	*R*^2^ = 0.07, Δ*R*^2^ = 0.05, *F*_(5, 215)_ = 3.34^**^					*R*^2^ = 0.08, Δ*R*^2^ = 0.04, *F*_(9, 211)_ = 1.99^*^								
	B	−0.13	−0.14	0.34	−0.12	−0.12	0.47	−0.15	0.36	−0.13	0.07	0.45	0.14	0.03	−0.05	−0.03
	*B*	−0.10	−0.11	0.12	−0.04	0.42	0.17	−0.12	0.13	−0.05	0.02	0.17	0.09	0.02	−0.04	−0.02
	*T*	−1.45	−1.64	1.28	−0.59	0.49	2.30^*^	−1.68^†^	1.35	−0.61	0.27	2.17^*^	1.00	0.30	−0.39	−0.23
Sympathy Physic		*R*^2^ = 0.02, *F*_(1, 219)_ = 3.21^†^	*R*^2^ = 0.13, Δ*R*^2^ = 0.11, *F*_(5, 216)_ = 6.45^**^					*R*^2^ = 0.17, Δ*R*^2^ = 0.14, *F*_(9, 211)_ = 4.75^**^								
	B	−2.22	−0.25	0.94	−0.05	−0.07	0.66	−0.28	0.94	−0.05	−0.07	0.66	0.46	0.08	−0.15	−0.23
	*B*	−0.12	−0.14	0.24	−0.01	−0.02	0.18	−0.16	0.24	−0.01	−0.02	0.18	0.23	0.04	−0.08	−0.12
	*T*	−1.79^†^	−2.12^*^	2.72^**^	−0.17	−0.22	2.46	−2.42	2.73^**^	−0.17	−0.22	2.46^*^	2.61^**^	0.57	−0.95	−1.64
Intervention Physic		*R*^2^ = 0.003, *F*_(1, 219)_ = 0.71	*R*^2^ = 0.04, Δ*R*^2^ = 0.01, *F*_(5, 215)_ = 1.61					*R*^2^ = 0.04, Δ*R*^2^ = 0.002, *F*_(9, 211)_ = 1.04								
	B	0.07	0.08	0.33	−0.35	0.06	0.20	0.09	0.35	−0.33	0.04	0.19	0.003	0.07	−0.08	−0.05
	*B*	0.06	0.07	0.12	−0.13	0.02	0.08	0.07	0.13	−0.12	0.01	0.07	0.002	0.05	−0.06	−0.04
	*T*	0.85	0.95	1.27	−1.97^*^	0.24	0.99	1.05	1.56	−1.61	0.15	0.94	0.02	0.63	−0.62	−0.49
Seriousness Verbal		*R*^2^ = 0.01, *F*_(1, 221)_ = 2.52	*R*^2^ = 0.06, Δ*R*^2^ = 0.04, *F*_(5, 217)_ = 2.66^*^					*R*^2^ = 0.08, Δ*R*^2^ = 0.04, *F*_(9, 211)_ = 1.92^†^								
	B	−0.19	−0.18	0.77	−0.38	−0.05	0.23	−0.19	0.84	−0.27	−0.17	0.18	0.15	0.23	−0.06	−0.17
	*B*	−0.11	−0.10	0.20	−0.10	−0.01	0.06	−0.11	0.22	−0.07	−0.05	0.05	0.08	0.12	−0.04	−0.09
	*T*	−1.59	−1.54	2.20^*^	−1.38	−0.17	0.85	−1.55	2.37^*^	−1.06	−0.51	0.67	0.81	1.53	−0.39	−1.21
Sympathy Verbal		*R*^2^ = 0.01, *F*_(1, 221)_ = 2.50	*R*^2^ = 0.14, Δ*R*^2^ = 0.12, *F*_(5, 217)_ = 6.83^**^					*R*^2^ = 0.16, Δ*R*^2^ = 0.12, *F*_(9, 211)_ = 4.35^**^								
	B	−0.17	−0.20	0.89	0.14	0.20	0.33	−0.20	0.97	0.12	0.07	0.26	0.27	0.11	−0.14	0.01
	*B*	−0.11	−0.12	0.26	0.04	0.06	0.10	−0.12	0.28	0.04	0.02	0.08	0.15	0.06	−0.09	0.004
	*T*	−1.6	−1.9^†^	2.90^**^	0.56	0.70	1.38	−1.85^†^	3.12^**^	0.50	0.24	1.09	1.96^*^	0.80	−0.99	0.06
Intervention Verbal		*R*^2^ = 0.00, *F*_(1, 211)_ = 0.01	*R*^2^ = 0.05, Δ*R*^2^ = 0.03, *F*_(5, 216)_ = 2.38					*R*^2^ = 0.06, Δ*R*^2^ = 0.02, *F*_(9, 212)_ = 1.58								
	B	−0.01	0.01	0.62	−0.62	−0.16	0.35	−0.002	0.60	−0.62	−0.16	0.35	−0.01	−0.01	0.02	−0.18
	β	−0.01	0.01	0.18	−0.19	−0.05	0.11	−0.001	0.18	−0.19	−0.05	0.11	−0.01	−0.01	0.01	−0.10
	*t*	−0.11	0.09	1.97^*^	−2.45^*^	−0.53	1.42	−0.02	1.88^*^	−2.45^*^	−0.53	1.42	−0.08	−0.10	0.15	−1.33
Seriousness Relational		*R*^2^ = 0.06, *F*_(1, 218)_ = 14.08^**^	*R*^2^ = 0.08, Δ*R*^2^ = 0.06, *F*_(5, 214)_ = 3.79^**^					*R*^2^ = 0.09, Δ*R*^2^ = 0.05, *F*_(9, 210)_ = 2.17^*^								
	B	−0.43	−0.42	0.51	−0.31	−0.36	0.29	−0.43	0.53	−0.29	−0.40	0.29	0.11	0.04	−0.07	−0.08
	β	−0.25	−0.24	0.14	−0.09	−0.10	−0.08	−0.25	0.14	−0.08	−0.11	0.08	0.06	0.02	−0.04	−0.04
	*t*	−3.75^**^	−3.64^**^	1.51	−1.15	−1.15	1.08	−3.57^**^	1.55	−1.10	−1.22	1.01	0.63	0.27	−0.40	−0.54
Sympathy Relational		*R*^2^ = 0.07, *F*_(1, 218)_ = 15.69^**^	*R*^2^ = 0.11, Δ*R*^2^ = 0.09, *F*_(5, 214)_ = 5.09^**^					*R*^2^ = 0.14, Δ*R*^2^ = 0.10, *F*_(9, 210)_ = 3.69^**^								
	B	−0.46	−0.48	0.55	0.05	0.01	0.30	−0.50	0.63	0.03	−0.15	0.25	0.37	0.16	−0.13	−0.13
	*B*	−0.26	−0.27	0.15	0.20	0.003	0.27	−0.29	0.17	0.01	−0.04	0.07	0.19	0.08	−0.07	−0.07
	*t*	−3.96^**^	−4.14^**^	1.61^†^	0.79	0.04	1.11	−4.26^**^	1.84^*^	0.13	−0.46	0.92	2.11^*^	1.06	−0.80	−0.89
Intervention Relational		*R*^2^ = 0.02, *F*_(1, 219)_ = 4.34^*^	*R*^2^ = 0.04, Δ*R*^2^ = 0.02, *F*_(5, 215)_ = 1.75					*R*^2^ = 0.07, Δ*R*^2^ = 0.03, *F*_(9, 211)_ = 1.61^†^								
	B	−0.23	−0.21	0.53	−0.37	−0.17	−0.15	−0.22	0.56	−0.29	−0.19	−0.11	0.25	0.02	−0.20	−0.26
	β	−0.14	−0.12	0.15	−0.11	−0.05	−0.04	−0.13	0.16	−0.09	−0.05	−0.03	0.14	0.01	−0.12	−0.14
	*t*	−2.08^*^	−1.82^†^	1.60	−1.74^†^	0.53	−0.58	−1.87^†^	1.70	−1.12	−0.59	−0.40	1.44	0.14	−1.29	−1.83

† *0.05 < p < 0.10*.

* *p < 0.05*.

** *p < 0.01*.

*Yos, Years of study; EC, Empathic concern; PD, Personal distress; PT, Perspective taking; FS, Fantasy*.

First, the results indicate that preservice teachers' years of study did not play any role in the three responses (seriousness perception, degree of empathy, or likelihood of intervention) to physical and verbal bullying (all *Fs* in model 1, 2, and 3 are greater than 0.05). However, the response to relational bullying was negatively predicted by the studying years; specifically, participants' years of study significantly and negatively predicted their seriousness perception to relational bullying, their degree of sympathy to relational bullying, *p* < 0.001, and their likelihood of intervention (see Table 3). This suggests that teachers with longer studying experience have decreasing responses to relational bullying, whereas the years of study does not play any role in preschool teachers' responses to physical or verbal bullying.

Second, we observed significant interactions between preservice teachers' empathic concern and their years of study on the scores of sympathy in responding to victims in all three bullying situations (also see Table 3). Subsequently, three simple slope analyses were conducted to examine the interactive effects. The participants were divided into high and low empathic concern groups according to the mean scores of empathic concern. Then we conducted three 2 (high empathic concern group vs. low empathic concern group) × 4 (years of study) variance analyses, with each of the teachers' sympathy for the victim in each of the physical, verbal, and relational bullying situations as the dependent variable.

The results suggest that teachers with high and low empathic concern show different studying years trends of sympathy for victims. Those with low trait empathic concern had significant decreases in their sympathy reaction to all three bullying situations. Specifically, for verbal bullying, the participants in their first studying year were significantly higher than those in their 4th year of study in their sympathy score: *F*_(3, 116)_ = 3.69, *p* = 0.01; for physical bullying, both the participants in their 1st and 3rd year of study scored higher than those in their 4th year of study; and for the relational bullying, the participants in their 1st year of study scored higher than the others: *F*_(3, 113)_ = 7.19, *p* < 0.001. For participants with high empathic concern, there was no significant difference in their degree of sympathy for victims in any of the three bullying situations: (*p*s > 0.05).

Third, Table 3 shows that the main effects of the participants' personal distress on seriousness perception and degree of sympathy were not significant in any of the bullying situations. However, personal distress negatively predicted the participants' likelihood of intervention: for physical bullying. That is, preservice teachers with a higher personal distress trait are significantly less likely to intervene and disrupt bullying behaviors.

## Discussion

The present study examined preservice preschool teachers' responses to bullying and the related roles of empathy dimensions. In accordance with the previous studies (e.g., Bauman and Del Rio, 2006), this study also underscore the importance of preschool teachers' ability to recognize, attend to, and intervene in relational bullying. Moreover, we explored the relationship between empathy and the reaction to bullying in the preservice preschool teachers. The results broaden our knowledge and understanding in two critical ways.

### Preservice teachers' responses to bullying behaviors

Preservice preschool teachers responded most strongly to physical bullying and less to relational bullying. This result is in accordance with research in primary and high schools (Bauman and Del Rio, 2006; Asimopoulos et al., 2014). Furthermore, when encountering more direct forms of bullying (e.g., physical bullying), preschool teachers made intervening steps prior to the seriousness perception and sympathy to the victim; however, when encountering more indirect bullying (e.g., relational) preschool teachers tend to give greater priority to sympathy over intervening. The underlying reasons may be different compared to teachers in other institutions such as primary or high schools. Unlike relational bullying among older children and adolescents, which is more subtle and ambiguous (Harachi et al., 1999; Bauman and Del Rio, 2006; Splett et al., 2015; Brion-Meisels and Garnett, 2016), it is simple, direct, and much easier to observe in preschool children. Therefore, teachers can easily tell when someone's feelings are hurt. It is possible that preschool teachers respond less to it because compared with physical and verbal bullying, which can be easily be judged as right or wrong, relational bullying is harder to “deal with.” It seems that relational bullying is a normative behavior (Brion-Meisels and Garnett, 2016), particularly for girls (Splett et al., 2015). In an interview study, teachers believed that the behavior of relational bullying were “normal human behavior” and were confused about the reasons why we have to intervene these behavior (Owens et al., 2000). Moreover, it is less likely to expressly forbidden by policy the behaviors in relational bullying, and teachers do not feel confident in such subjective judgments or the method to deal with this kind of bullying, which may contribute to their reluctance to respond (Ostrov et al., 2015). Preschool teachers require more professional knowledge and concrete strategies to correctly and appropriately address relational bullying.

We did not observe significant changes in the preservice teachers' physical and verbal bullying among the different years of study, but their reactions to relational bullying significantly decreased with more years of education. This result is counterintuitive and is contrary to our initial hypothesis since preschool teachers' abilities to recognize and address relational bullying increase with their years of study and more practice (Bauman and Del Rio, 2006). We can explain this result from the perspectives of both the preschoolers and teachers. Although relational bullying in younger preschooler is simple and direct, as preschoolers' cognition and social interaction develop, their relational bullying becomes more ambiguous and cunning. It is more difficult to detect relational bullying among older children. Because the harms of relational bullying cannot be observed directly, the teacher must inferred them from the victim's behaviors or his/her own experience. Second, from the teachers' perspective, detecting relational bullying requires more skill and cognitive resources. Furthermore, relational bullying is rapidly increasing, possibly due to preschool teachers' decreased responses to this subtype (Bauman and Del Rio, 2006). Perhaps because of all these reasons from the preschoolers and the preschool teachers, the preservice teachers' responses to relational bullying show a trend of decreasing.

### Preservice teachers' trait empathy

We found that the four components of empathy show distinct change trajectories in preservice preschool teachers. Except fantasy keep stable, the other three components all changed significantly across the four years. While empathic concerns and perspective taking similarly decrease from the 1st−3rd year of study and increase in the 4th year of study, personal distress shows the opposite pattern of increasing from the 1st and peaking in the 3rd year of study. It is interesting that the 3rd year of study is the turning point for both components as the same phenomenon has been described for medical students (Neumann et al., 2011). We hypothesize that increased practice in the 3rd year may contribute to this change.

Considerable research suggests that medical staff and student empathic concern decreases with greater medical experience (Neumann et al., 2011; Teng et al., 2018), and some researchers believe the underlying mechanisms are dehumanization and detachment, which are indexes of job burnout (Haslam and Loughnan, 2014). Based on daily observation, it is undeniable that student preschool teachers usually pay too much attention to children's feeling or are over-engaged in the relationships at the beginning of their studies, and this can be stressful. Some researchers have pointed out that preschool teacher jobs are typical emotional labor, of which emotional engagement is a core part (Philipp and Schüpbach, 2010). Empathy that is too strong or lasts too long may increase the risk of emotional exhaustion (Wróbel, 2013; Tabaj et al., 2015; White et al., 2015). Near constant exposure to children's emotions could decrease preschool teachers' sensitivity to emotional cues. The changes that took place in the preschool teachers may reflect a protective mechanism. From this perspective, this result also supports the notion that adult personality development is affected by career and professional factors (Rushton et al., 2007). The present research did not provide enough evidence to support burnout as the cause for changes in preschool teachers' trait empathy; further studies are needed to clarify the why teachers' show less reaction to relational bullying at the end of their studies.

### Relationship between trait empathy and reaction to bullying in preschool teachers

We also examined the relationship between preservice preschool teachers' responses to bullying and the four empathy components. First, there are complex relationship between the two emotional components of empathy. Second, to our surprise, the two cognitive components of empathy do not have any meaningful relationship with various index of the preschool teachers' responses to bullying.

First, our study revealed complex relationship between the two components of emotional empathy and the indexes of the preservice preschool teachers' responses to bullying. On the one hand, there were significant differences in sympathy for the victim between preservice teachers with low and high empathy. On the other hand, while personal distress did not correlate with the severity evaluation or sympathy for victims, it significantly predicted the preservice teachers' likelihood of intervening in bullying. Thus, even though they consider bullying as serious and feel sympathy for the victim, they may not take steps to stop it. We also found that preschool teachers with high personal distress are less likely to intervene in bullying.

These results can be partially explained by the theory about the relationship between empathy and prosocial behavior since preschool teaching is a typical helping profession and teachers' interventions into bullying behavior is similar to prosocial behavior. According to Eisenberg et al. (1989), personal distress and empathic concern have different influences on prosocial behavior. Extending the metaphor, the two components would also have different impacts on teachers' interventions into bullying. First, empathic concern is the real motivation for prosocial behavior, as empathic concern is expected to lead to the other-oriented, altruistic behavior and aims to alleviate the other's distress. Teachers who experience empathic concern tend to deal with the children's bullying even if they have the choice to escape (see Batson, 1987). Individuals with high empathic concern may have more resistance to exposure to children's emotions and can maintain their empathy (Lamothe et al., 2014). In contrast, personal distress is an egocentric motivation, and people who experience personal distress always help others to relieve his or her own uncomfortable internal state. Preschool teachers dealing with bullying have the characteristic of prosocial behavior; therefore, it is not surprising that teachers with high personal distress are less likely to deal with bullying, even though they can judge it as very serious and feel sorry for the victims. In preschool situations, teachers experiencing personal distress are expected to intervene in bullying primarily when they cannot easily escape and have no choice.

However, in the present study, the two components of the empathy only explain a modest percentage of the preschool teachers' responses to bullying. This finding was in line with the research of Eisenberg et al. (1989), which found that while it is often assumed that empathic concern plays an essential role to motivate prosocial behavior when in response to another's distress, the relationship between them is often modest. A possible reason for the limited association between empathy and responses to bullying may be the effect of moderating variables. Recent research suggested that the tendency to reappraise, a typical emotional regulation strategies, may play an important role in the relationship between empathy and prosocial behavior (Lockwood et al., 2014), therefore it is also possible that the individual difference in emotion regulation is a deeper potential factor underlying the relationship between empathy and response to bullying.

Second, contrary to our initial hypothesis, our study found neither of the two cognitive empathy, especially perspective-taking which is widely defined as a fundamental social skill required for successful social interaction (Underwood and Moore, 1982; Krauss and Fussell, 2011), have any meaningful relationship with the preservice preschool teachers' responses to bullying. Research concerning social power and social cognition may help us understand the zero relationship between cognitive empathy and teachers' responses to bullying. Social power is associated with feeling in control over one's environment and being the decision maker in relationships, however perspective-taking is a strategy used to infer the actions of others (Blader et al., 2016). Multiple theories and research indicated that social power may decreases one's perspective-taking (Galinsky et al., 2016). Compared with the children, the preschool teachers are obviously advantaged in social power, therefore it is comprehensible that the teachers tend to understand the situation and judge its consequences on the basis of his or her own experience, with less motivation taking a step into the children's minds.

### Values, limitations, and future directions

This study was an important attempt to integrate the potential factors of teachers' reactions to bullying. The results provide valuable insight into how empathy is related to prosocial behavior (bullying intervention) in certain professional environment social competences. Furthermore, the Chinese sample of the present study broadens our understanding about this important problem by adding evidence in non-Western cultures. Cross-culture studies are needed to compare directly the differences between Chinese and Western teachers in the future.

This study has several limitations that must be mentioned. First, the cross-sectional design is unable to provide a causal explanation with regard to how empathy influences preservice teachers' reaction to bullying. Second, the bullying reactions and empathy scores were all based on self-reports. Although we utilized the most widely used measures of empathy and teachers' responses to bullying, societal stereotypes could bias self-report evaluation. Future studies should include implicit measures or behavior indexes, and a longitudinal design or intervention research would be a better way to identify the potential mechanism(s) underlying teacher responses to bullying.

## Conclusions and practical implications

Our results indicate that preservice preschool teachers generally have the strongest response to physical bullying but respond less to relational bullying; moreover, their responses to relational bullying showed a decreasing trend for students in more advanced grades. In general, their responses to bullying subtypes showed different patterns. Notably, we found that the three components of empathy, empathic concern, personal distress, and perspective taking, exhibited different trajectories over the educational period of preservice teachers. These dimensions played different roles in the preservice teachers' responses to bullying. Perhaps it is due to the imbalance of power between the teacher and the children, when dealing with bullying the teachers were less motivated to take the perspective of the children. While cognitive empathy do not bare any regular correlation with preschool teachers' responses to bully, the two dimensions of emotional empathy, those are empathic concern and personal distress, showed complex relationship with teachers' responses to bullying. Empathic concern moderated the relationship between preservice teachers' years of study and their response to all types of bullying. Individuals with high empathic concern maintained stable response levels with all forms of bullying, showed significantly more sympathy to the bullying victim, and had similar response levels across the year of study. Among those with lower empathic concern, their sympathy with the bullying significantly decreased with more advanced years of study. On the other hand, personal distress negatively predicted the participants' likelihood of intervening in all types of bullying.

First, we found that relational bullying tended to be regarded by preservice teachers as less important than more direct forms of bullying. However, research indicates that negligence may be harmful to both the bully and their victims. Relational bullying is related to more emotional (Casey-Cannon et al., 2001), social (Murray-Close and Crick, 2006), and behavioral (Murray-Close and Crick, 2006) problems. Our finding suggests that more attention should be given to relational bullying to avoid marginalizing the concerns of relationally victimized children. These results also call for the development and implementation of niche-targeting teacher education programs. Both in preservice or in-service teachers, there is currently a lack of comprehensive training to prevent or intervene bullying in general, the training to prevent or intervene relational training are in even more serous lack (Holt and Keyes, 2004). To be aware of and address relational bullying, the teacher must have sufficient background knowledge to recognize it; they should also be required to have the skills to deal with it. This is especially important in preschool settings, as relational bullying is just emerging in this period and is not always obvious. Accordingly, teacher training is necessary and must contain both knowledge and skills for addressing relational bullying at preschool. Offering such teacher education at universities and teachers' colleges should be a high priority.

Second, our results underscore the importance of empathy in teachers' responding to bullying. Empathy, containing both cognitive and emotional components, is an essential trait for working with preschool children, so it should be an integral part of every teacher's personality. It may be a useful index to assess during preschool teacher recruitment and education. It seems that individuals with higher empathic concern and lower personal distress are more likely to adopt the job demands of preschool teachers. This study also suggest that preschool teachers should take the children's perspective more proactively, and pay more attention to the children's emotion and thoughts. Finally, our findings indicate that teacher education should include instruction on regulating empathy to avoid decreases in this trait over time.

## Ethics statement

The material has not been published in whole or in part elsewhere. The paper is not for publication elsewhere. All authors have been personally and actively involved in substantive work leading to the report, and will hold themselves jointly and individually responsible for its content. All relevant ethical safeguards have been met in relation to participants protection. I testify to the accuracy of the above on behalf of all the authors.

## Author contributions

HH was in charge of study design and manuscript writing, and also help in data collection. YL was in charge of statistical analysis and helped in data collection. YC was in charge of data collection. Moreover, the three authors all contributed in the data interpretation and the writing of Discussion.

### Conflict of interest statement

The authors declare that the research was conducted in the absence of any commercial or financial relationships that could be construed as a potential conflict of interest.
